# Enzyme Inhibitory Potential of *Ligustrum lucidum* Aiton Berries

**DOI:** 10.3390/molecules24071283

**Published:** 2019-04-02

**Authors:** Vanessa B. Paula, Teresa Delgado, Maria Graça Campos, Ofélia Anjos, Leticia M. Estevinho

**Affiliations:** 1 Escola Superior Agrária, Instituto Politécnico de Bragança, 5300-253 Bragança, Portugal; vanessapaula@ipb.pt; 2 Centro de Biotecnologia de Plantas da Beira Interior, 6001-909 Castelo Branco, Portugal; teresadelgado86@hotmail.com (T.D.); ofelia@ipcb.pt (O.A.); 3 Observatório de Interações Planta-Medicamento (OIPM)|Faculdade de Farmácia, Universidade de Coimbra, Campus das Ciências da Saúde, 3000-548 Coimbra, Portugal; mgcampos@ff.uc.pt; 4 Centro de Química de Coimbra (CQC, FCT Unidade 313) (FCTUC) Universidade de Coimbra, 3004-535 Coimbra, Portugal; 5 Instituto Politécnico de Castelo Branco, 6001-909 Castelo Branco, Portugal; 6 Centro de Estudos Florestais, Instituto Superior de Agronomia, Universidade Lisboa, 1349-017 Lisboa, Portugal; 7 Centro de Investigação de Montanha (CIMO), Instituto Politécnico de Bragança, 5300-252 Bragança, Portugal

**Keywords:** *Ligustrum lucidum* Aiton berries, diabetes, Alzheimer’s disease, enzyme inhibitory potential

## Abstract

*Ligustrum lucidum* Aiton and its berries have been used in Chinese traditional medicine for around two thousand years. In the present study, *L. lucidium* berries harvested in two regions of Portugal were studied. Haemolytic activity and inhibition of oxidative haemolysis as well as the enzyme inhibitory activities (α-amylase enzyme and acetylcholinesterase) were assessed. Results suggest that the different biological activities varied according to the region where samples were collected. Results demonstrated that the sample obtained from region R1 was the most efficient extract for all parameters evaluated, presenting the lowest values of IC_50_, 10.67 ± 0.46 μg/mL for the inhibition of erythrocyte oxidative haemolysis, 58.28 ± 3.77 μg/mL for the α-amylase enzyme and 67.67 ± 2.10 μg/mL for the acetylcholinesterase inhibition. *L. Lucidum* berries may be an interesting source of compounds for use in the development of the therapeutic armamentarium for diseases where enzymatic disruption is believed to play a role.

## 1. Introduction

*Ligustrum lucidum* Aiton is a plant that naturally occurs in hot and humid climates, namely in the east, south and south-west of China, South Korea and India [1]. However, it has been reported to be one of the most invasive plant species in the world, having global relevance. In ancient times, their leaves and berries were harvested, dried and applied in the traditional medicine for several purposes, such as premature menopause, blurred vision, cataracts, tinnitus, rheumatic pains, palpitations, backache and insomnia [2]. Several studies were performed with the aim of characterizing the composition of *Ligustrum lucidum* fruits, revealing the presence of triterpenoids, iridoids, flavones, phenolic glucosides and other less abundant components, among which are polysaccharides, amino acids, fatty acids, volatile components, pigments and other minor elements [3].

Different biological activities from its berries have been studied in recent years, namely anti-inflammatory, antitumour, antioxidant, and antiviral activities [4,5,6]. However, as far as the authors know, there are no studies regarding the enzyme inhibitory potential of the berries and very little information is available regarding *L. lucidum* produced in Europe, particularly in Portugal. Changes in the enzymatic activity and pathways are involved in the pathogenic mechanisms of several diseases: the aberrant increase activity of acetylcholinesterase is associated with Alzheimer’s disease, while higher activity of the digestive enzyme α-amylase plays a role in obesity and type 2 Diabetes mellitus [7]. 

Therefore, the aim of the present study was to evaluate the enzyme (α-amylase and acetylcholinesterase) inhibitory potential as well as the haemolytic activity and inhibition of oxidative haemolysis of *L. lucidum* berries harvested in two different Portuguese regions [Lisbon (R1) e Castelo Branco (R2)]. These two regions are representative in terms of distribution and present different edaphoclimate conditions, and it was decided to evaluate the effect of region on several enzymes because we wanted to obtain a comprehensive view of the possible beneficial effects of this plant on human health.

## 2. Results and Discussion

### 2.1. Hemolytic Activity and Inhibition of Oxidative Hemolysis

The hemolytic activity of 50% ethanol *L. lucidum* extract and its ability to protect erythrocytes against hemolysis induced by the oxidizing agent 2,2′-azobis (2-methylpropionamidine) dihydrochloride (AAPH) were evaluated.

**Figure 1:**
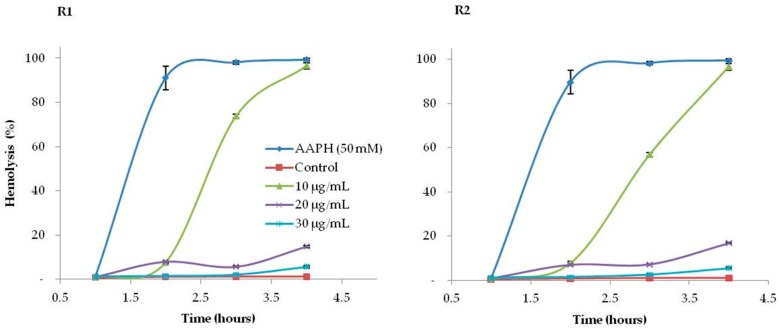
Hemolytic activity of *L. lucidum* berries, from the two Portuguese regions (R1—Lisbon and R2—Castelo Branco), incubated with erythrocytes and 0.9% NaCl solution for 240 min, without the presence of 2,2′-azobis (2-methylpropionamidine) (AAPH). The control consists of erythrocytes incubated only with 0.9% NaCl solution.

**Figure 2:**
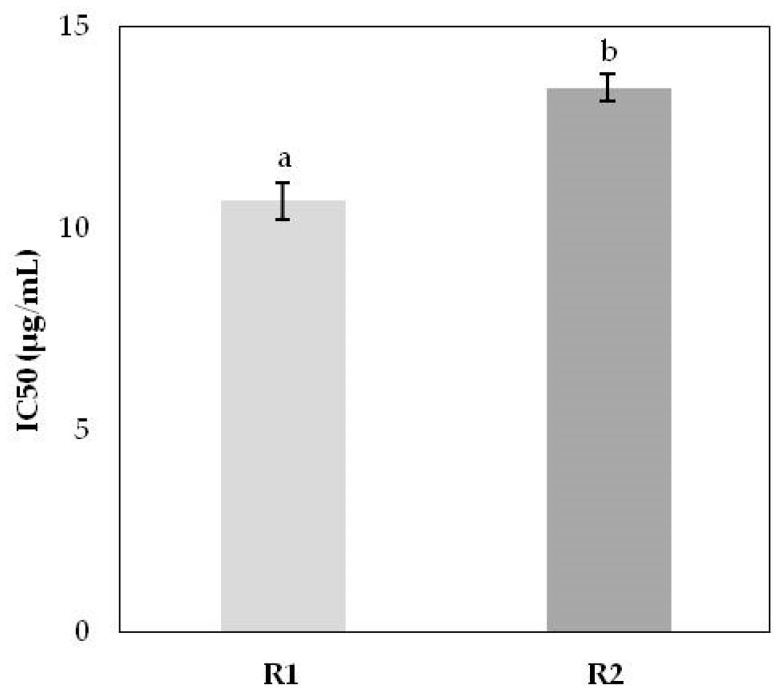
Antioxidant activity of *L. lucidum* extracts in vivo with 50% ethanol, from the two Portuguese regions in study (R1—Lisbon and R2—Castelo Branco).

The results obtained for the inhibition of erythrocyte oxidative hemolysis induced by *L. lucidum* extracts are presented in Figure 1 and Figure 2. It was observed that erythrocytes incubated with the highest concentrations of extracts or ascorbic acid practically did not undergo hemolysis. However, the addition of AAPH induced time-dependent hemolysis. The extracts significantly protected the erythrocytes against damage caused by the oxidizing agent with IC_50_ values after 3 h of incubation ranging from 10.67 ± 0.46 μg/mL (R1) to 13.48 ± 0.23 μg/mL (R2).

Inhibition induced by ascorbic acid (AA, 99.7%) (Merck and Riedel-de Haën), positive control, was lower (IC_50_ = 45.05 ± 0.23 μg/mL) and different from that obtained in the presence of the extracts.

### 2.2. Enzyme Inhibitory Activities

The authors evaluated the effect of *L. lucidum* extracts in the enzymatic activity of α-amylase and acetylcholinesterase in order to obtain a comprehensive idea of the possible beneficial effects of these extracts in two very important groups of diseases where those enzymes play a role—neurodegenerative and metabolic.

#### 2.2.1. Inhibition of α-Amylase

**Figure 3:**
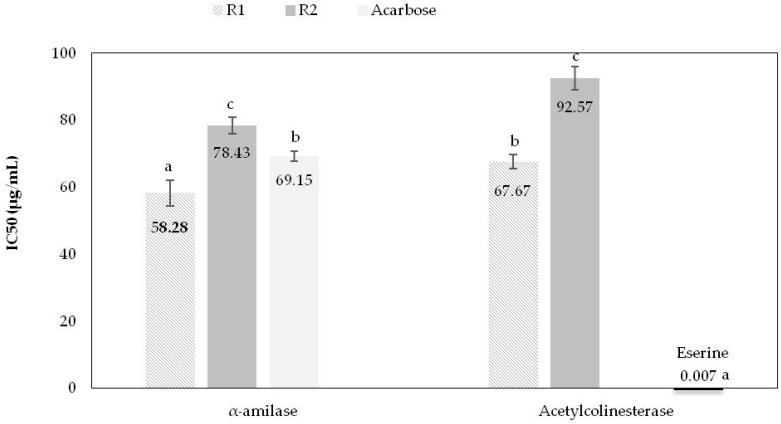
Enzyme inhibitory activities of *L. lucidum* berry extracts (mean values and standard deviations) from the two Portuguese regions in study (R1—Lisbon and R2—Castelo Branco). Mean ± SD. Different small letter (a–c) superscripts on the same column are significantly different (*p* < 0.05).

The inhibition of α-amylase may delay carbohydrate digestion and glucose uptake with a consequent reduction in postprandial blood glucose level in diabetic patients [8]. The study of this effect is relevant, considering that more than 40% of the Portuguese population has Diabetes mellitus or intermediate hyperglycemia. In the present study, it was found that all extracts inhibited α-amylase. The extract from Region 1 induced the highest inhibition (IC_50_ of 58.28 ± 3.77 μg/mL), even below acarbose, the positive control. The extracts from R2 were less effective (IC_50_ of 78.43 ± 2.50 μg/mL) but not statistically different from the value obtained for the control (69.15 ± 1.49 μg/mL) (Figure 3). 

The concentrations of *L. lucidum* extracts required to induce 50% inhibition of α-amylase were 10-fold lower than those reported by Kumar et al. [9] in medicinal plant extracts from India (IC_50_ = 562.18 ± 5.98 μg/mL).

#### 2.2.2. Inhibition of Acetylcholinesterase

Currently, Alzheimer’s disease is the leading cause of dementia and with the increasing ageing of the population, its prevalence is expected to triple over the next decades. As such, the development of strategies that slow or halt Alzheimer’s disease progression is imperative for improving patients’ quality of life and to reduce the attributable health care costs [10]. taking this into account, the effect of *L. lucidum* extracts on the inhibition of acetylcholinesterase was evaluated. 

In this study, it was observed that R1 (IC_50_ = 67.67 ± 2.10 μg/mL) had the highest acetylcholinesterase inhibition, as described in the previous assay. Higher IC_50_ values were obtained for R2 samples, meaning a lower acetylcholinesterase inhibition (Figure 3).

The inhibition observed for the control, Eserine, differed significantly from that obtained using extracts. 

## 3. Materials and Methods 

### 3.1. Plant Samples

The broad-leaf privet berries (*L. lucidum* A.) used in this study were acquired in Portugal in December 2016 and frozen at −20 °C until the extraction procedures were carried out. In order to study the effect of different climatic conditions on the characteristics and biological properties of the plants, samples from two regions (Lisbon, R1; and Castelo Branco, R2) were analysed and compared.

### 3.2. Extraction Conditions

The extraction procedure was performed as previously described [5]. In the previous work, several extraction methods were tested in order to obtain the extract with the highest antioxidant power. Three different solvents were tested: boiling water (45 min); 100% ethanol (shacked 6, 12 and 24 h) and 50% ethanol–water (*v*/*v*) (shacked 6, 12 and 24 h). All experiments were performed in triplicate, at room temperature in the dark. MiliQ-water was used for the extraction and 96% ethanol was purchased from Merck. The extract presenting the highest antioxidant activity was then prepared with 50% ethanol following 15 h of stirring at 120 rpm, at room temperature. Therefore, in this study, the extracts were prepared by mixing 20.5 g of plant with 50 mL of solvent. After this, the samples were centrifuged at 5000 rpm for 10 min and the supernatant was transferred to a vial and stored at −20 °C until further analysis. All extractions were carried out in duplicate, and all further measurements and analysis were then carried out in triplicate. The final extract concentration was 50 mg/mL.

### 3.3. Antioxidant Assays in Erythrocytes

#### 3.3.1. Preparation of Erythrocyte Suspensions

Peripheral blood samples (20 mL) were collected via jugular venipuncture from four sheep, randomly chosen, allocated at the Polytechnic Institute of Bragança. Sheep were 2–3 years old at the time of blood collection and were carefully examined by veterinarians in order to exclude the presence of any signs of disease. All procedures were in agreement with the guidelines of the EU Directive 2010/63/EU (European Union, 2010) for the protection of animals used for experimental and other scientific purposes and were approved by local Animal Care and Use Committee.

The blood was placed in tubes containing sodium citrate. Tubes were then centrifuged at 700 g for 10 min and the blood plasma and leukocyte layer were discarded.

The erythrocytes were washed three times with 0.9% sodium chloride (NaCl—Sigma (Madrid, Spain)) solution, and then a 10% erythrocyte suspension in 0.9% NaCl solution was prepared for the inhibition assays of oxidative haemolysis, determined using the method described by Campos et al. [11].

#### 3.3.2. Inhibition of Oxidative Haemolysis

The cells were preincubated at 37 °C for 30 min in the presence of different concentrations of the extract (10 to 60 μg extract/mL). After this period, 50 mM AAPH (2,2′-azobis-2-amidinopropane Sigma (Madrid, Spain)) solution was added. This reaction mixture was shaken gently while being incubated at 37 °C for 4 h. In all experiments, a negative control (erythrocytes in PBS—Sigma (Madrid, Spain) (erythrocytes in a phosphate buffered saline (PBS) of 150 mmol/L NaCl, 1.9 mmol/L NaH2PO4,8.1 mmol/L Na2HPO4, pH 7.4)) and extract controls (erythrocytes in PBS with each extract) were used. The extent of haemolysis was determined spectrophotometrically (Jenway) according to a method reported before [12]. Briefly, aliquots of the reaction mixture were taken out at each hour of the 4 h of incubation, diluted with saline (0.9%NaCl solution), and centrifuged at 4000 rpm for 10 min to separate the erythrocytes. The percentage of haemolysis was determined by measuring the absorbance of the supernatant (A) at 545 nm and compared with that of complete haemolysis (B) by treating an aliquot with the same volume of the reaction mixture with distilled water. The haemolysis percentage was calculated using the formula:

(1) $$Hemolysis\left(\%\right)=\left(\frac{Abs\;sample}{Abs\;total\;hemolysis}\right)\;\times 100$$

Assays were performed in triplicate, and ascorbic acid (AA, 99.7%) was used as the control.

### 3.4. Acetylcholinesterase Inhibition

The inhibition of acetylcholinesterase was evaluated spectrophotometrically following the method of Ellman et al. [13] with slight modifications. Acetylcholinesterase of *Electrophorus electricus* (electric ceel Type-VI-S, Sigma Chemical Co, St. Louis, MO, USA) was used. The acetylcholinesterase activity was quantified using 15mM 5,5′-dithiobis-(2-nitrobenzoic acid) (DTNB, Sigma Chemical Co, St. Louis, MO, USA). The hydrolysis of acetylcholine iodide was monitored by the formation the anion 5-tio-2-methyl nitrobenzoate (yellow colour), measured at 412 nm. The percentage of inhibition was determined by comparing the rates of reaction of samples (final concentrations of 1000, 750, 500, 250, 50, 10, 1.5 μg/mL) with those of the blank (ethanol in phosphate buffer (Merck and Riedel-de Haën, 0.2 M, pH = 8) using the following formula, where *E* is the activity of enzyme without test sample and *S* is the activity of enzyme with the sample under analysis:

(2) $$\frac{E-S}{E}\times 100$$

The extract concentration providing 50% inhibition (IC_50_) was calculated by interpolation from the graph of I% against extract concentration, following linear regression analysis with 95% of confidence level using the standard curve built with serine (Sigma-Aldrich (Madrid, Spain)) as reference.

### 3.5. α-Amylase Inhibition

The assessment of α-amylase inhibition was performed as reported by Thilagam et al. [14] using starch azure (Sigma Chemical Co, St. Louis, MO, USA) as substrate. It was dissolved (2 g) in buffer Tris 50 mM-HCl (Merck, Darmstadt, Germany) (pH 6.9) containing 10 mM CaCl_2_ (Merck and Riedel-de Haën) the mixture was boiled at 100 °C for 5 min and preincubated at 37 °C for 10 min. Afterwards, the sample dissolved in DMSO (Sigma-Aldrich, Madrid, Spain). (50%), at final concentrations of 1250, 1000, 750, 500, 250, 50 and 10 μg/mL, was added to each test. Then, 0.1 mL of α-amylase from pig pancreas (Sigma, A-6255; 2.0 U/mL; 50 mM Tri-HCl) was added. The samples were then incubated at 37 °C for 10 min, after the reaction was stopped by adding 0.5 mL of acetic acid at 50%. The mixture was centrifuged at 2000 rpm for 5 min and the absorbance of the supernatant was measured at 595 nm. Acarbose (Sigma-Aldrich, Madrid, Spain) was used as the positive control. α-amylase inhibition was calculated using the following equation:

(3) $$I\left(\%\right)=100-\left(\frac{B-b}{A-a}\right)\times 100$$

where *A* is the activity without inhibitor; *a* is the negative control without inhibitor; *B* is the activity with inhibitor; and *b* is the negative control with inhibitor. 

The extract concentration providing 50% inhibition (IC_50_) was calculated by interpolation from the graph of I% against extract concentration, following linear regression analysis with 95% of confidence level.

### 3.6. Statistical Analysis

To compare the values obtained, a Least Significant Difference (LSD) test post-hoc test with 95% confidence was applied to the corresponding variables. This test allows making a direct comparison between two means from two individual groups; any difference larger than the LSD is considered a significant result. The statistical analysis was performed on Statistica 13.0 software (Dell Software, Round Rock, TX, USA). 

## 4. Conclusions

Extracts from *L. lucidum* berries (prepared with 50% ethanol) from region R1 had a significantly superior inhibitory activity for both α-amylase and acetylcholinesterase, suggesting that the biological activities of *L. lucidum* berries appear to depend on their place of origin.

The interesting potential of the studied extracts to inhibit α-amylase and acetylcholinesterase enzymes suggests that these extracts (or isolated compounds) may be useful agents for controlling disorders where such enzymes play a role, although our results are still very preliminary. Indeed, plant extracts contain a complex composition profile and its biological effect cannot be attributed to one single compound. Further studies may then be performed in order to assess the absorption rate of the compounds available in these extracts and whether these are able to cross the blood–brain barrier.
